# Lithophagy Prolongs Voluntary Dives in American alligators (*Alligator mississippiensis*)

**DOI:** 10.1093/iob/oby008

**Published:** 2019-01-02

**Authors:** T J Uriona, M Lyon, C G Farmer

**Affiliations:** Trinity College Dublin, Dublin 2, Ireland; Department of Biology, University of Utah, Salt Lake City, UT 84112, USA

## Abstract

Many vertebrates ingest stones, but the function of this behavior is not fully understood. We tested the hypothesis that lithophagy increases the duration of voluntary dives in juvenile American alligators (*Alligator mississippiensis*). After ingestion of granite stones equivalent to 2.5% of body weight, the average duration of dives increased by 88% and the maximum duration increased by 117%. These data are consistent with the hypothesis that gastroliths serve to increase specific gravity, and that the animals compensate by increasing lung volume, thereby diving with larger stores of pulmonary oxygen.

## Background

Stones are found in the gastrointestinal tracts of many organisms, but the function of lithophagy in many lineages is unknown (Wings 2007). Vertebrates may ingest rocks by accident, or with the intent to grind food, as a source of minerals, or to rid themselves of parasites. In aquatic lineages these stones may serve as ballast, or to increase specific gravity (Wings 2007). There are few direct experimental tests of these functions, and most tests have focused on the role gastroliths play in trituration of ingesta in terrestrial lineages (Wings 2007). For example, a study of gastrolith mass and shape in ostriches concluded that a gastric mill evolved along the avian stem lineage (Wings and Sander 2007). Fewer experimental data are available for aquatic lineages, although the hypothesis that gastroliths serve to increase specific gravity is well established. Indeed, South American natives believed crocodylians swallowed stones “to augment their weight” (von Humbolt 1852). A comparative analysis of the distribution of gastroliths in both extant and extinct species concluded that, in marine tetrapods, gastroliths serve a role in regulating buoyancy rather than food processing (Taylor 1993). Because most rock has a greater specific gravity than animal tissue, ingestion of gastroliths decreases buoyancy if lung volume is not increased to compensate. This could aid in dragging struggling prey underwater, or facilitate maneuverability underwater—especially the capacity to stay still at the bottom in a current. Lithophagy could also allow the animals to increase their lung volume without becoming too buoyant, and thereby increase the store of oxygen during dives (Cott 1961; Seymour 1982; Taylor 1993; Grigg and Kirshner 2015). Seymour hypothesized that gastroliths would increase lung volume by about 12% in crocodiles (Seymour 1982). Henderson (2003) modeled the effects of gastroliths on buoyancy and stability in adult alligators and concluded these effects are inconsequential, and that specific gravity would be more easily adjusted with lung volume. Kirshner (1985) showed that crocodiles defend a particular level of buoyancy by increasing lung volume to compensate for an added gastrolith load, and that the animals are usually negatively buoyant with an average specific gravity of 1.028. Similarly, a small, compensatory change in lung volume will affect oxygen stores in alligators, and so even a small gastrolith load could affect the duration of dives.

The results of previous studies provide mixed support for the hypothesis that gastroliths extend the duration of dives. When steel ball bearings equivalent to 1% of body weight were placed into the stomachs of juvenile, saltwater crocodiles (*C. porosus*), the animals compensated by increasing the volume of air in the lungs by about 20% (Grigg and Kirshner 2015). When floats were attached to the animals, they reduced pulmonary volume to restore a desired buoyancy, although compensation was less complete in the latter case (Grigg and Kirshner 2015). However, these experimentally induced changes in pulmonary volume did not change the time the animals submerged voluntarily (Kirshner 1985; Grigg and Gans 1993; Grigg and Kirshner 2015). In freshwater turtles (*Trachemys scripta elegans*), external weights or floats added to the shells caused them to adjust pulmonary volume to compensate for the change in specific gravity (Jackson 1969, 1971), and added weights prolonged voluntary dives in green turtles (*Chelonia mydas*) (Hays et al. 2004). Experimentally induced changes in specific gravity with the types of food eaten affected the duration of voluntary dives in alligators (Uriona et al. 2009), suggesting the animals changed lung volume to compensate for the change in specific gravity associated with eating either negatively or positively buoyant food, and thereby triggered changes in the duration of dives. In summary, although the hypothesis that lithophagy in crocodylians prolongs dives is plausible, there are few direct experimental tests of this hypothesis, and the results of the most direct test did not support the hypothesis (Kirshner 1985; Grigg and Kirshner 2015). In this study, we tested the hypotheses that ingestion of gastroliths in juvenile alligators would increase the duration of voluntary dives by comparing the time animals spent submerged before and after ingesting granite gastroliths weighing 2.5% of their body weight.

## Methods

Seven juvenile American alligators (*Alligator mississippiensis* Daudin 1801) were obtained from the Rockefeller Wildlife Refuge (Louisiana, USA) and transported by land to Salt Lake City, UT, USA, where the experiments were conducted. Prior to entering the experiments, gastric lavage was used to remove any gastroliths that the animals had ingested while at the refuge. Radiographs were taken to ensure all gastroliths had been removed. The animals (*N* = 7; mean mass ±s.e. =379.3 g ± 37) were housed in a room maintained at 25°C, had access to basking platforms, and experienced a light:dark cycle of 12:12 h. They were fed ad lib on Mazuri^®^ crocodylian diet (PMI Nutrition International, Brentwood, MO, USA) and fasted 48 h before being placed in the diving arena.

To measure the duration of dives, animals were removed from their housing and placed in a diving arena maintained at 25°C. The arena was a glass 284 L aquarium (122 cm long, 45 cm wide, 61 cm high), which was filled three quarters full with water, producing a depth of about 46 cm. All observations were made by the same observer at a similar time of day. The observations were recorded with a stop watch. The animals were observed for 30 min, but the time spent diving during this introductory period was not recorded. Data collection then commenced and continued until seven dives lasting more than 1 min were recorded. This procedure was repeated on two separate days until 21 dives had been recorded for each animal.

After collection of this set of data, the animals were left overnight in cages with the appropriate number of gastroliths allocated to each cage according to the body mass of the animal. Granite stones (no bigger than 10 mm in diameter) totaling 2.5% of body mass were placed into each aquarium, and animals left undisturbed. Four of the animals voluntarily ingested all of the stones. For the three animals that consumed only a portion of the stones, the remaining stones were gently placed in the back of the mouth (caudad to the *velum palatinum*) along with approximately 1 cc of water, and the ventral side of the throat lightly stroked to stimulate swallowing and peristalsis. All animals retained the stones. After ingesting the stones, the diving protocol described previously was repeated until 21 dives had been measured on each of the animals. Dives lasting under 1 min were not included in the analysis because they consisted primarily of swimming. The effects of gastroliths on the time spent underwater were evaluated using a repeated measures analysis of variance conducted with Statistix 8.1 (*P* < 0.05 considered significant).

The specific gravity of one additional, deceased juvenile specimen was estimated by comparing the following measurements: (1) weight of the specimen out of the water, and (2) weight of the animal while submerged, but after compression of the thorax to expel air from the lungs.

## Results

**Fig. 1:**
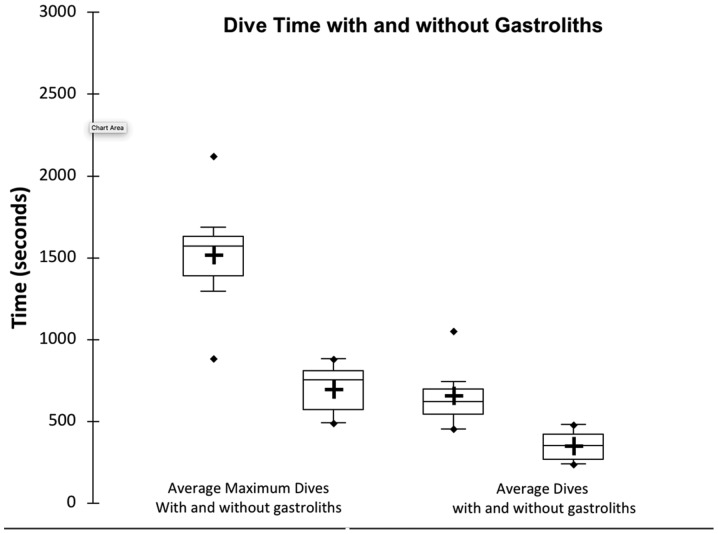
Box and whisker plot of mean duration of dives, and of the mean of the maximal dives, with and without gastroliths. Boxes, interquartile range; whiskers, the lower and upper quartile; + mean; − median line, ♦ shortest and longest mean dives or shortest and longest maximal dive.

The presence of gastroliths significantly prolonged the average duration of the dives as well as the duration of the average of the maximum dives (Fig. 1). Without gastroliths, the average period the animals spent submerged was 351 ± 184 s (mean ± SD, ∼5.9 min), which was significantly less than the average period when diving with gastroliths, 660 ± 372 s (mean ± SD, ∼11 min). Thus, ingestion of gastroliths increased the duration of the dives by 88%. The duration of the average of the maximum dives also increased with the presence of gastroliths by 117%, from 699 ± 151 s (mean ± SD, ∼11.7 min) without gastroliths to 1518 ± 375 s (mean ± SD, ∼25.3 min) with gastroliths. All the alligators increased the duration of the maximum dive by 305 s or more when given the gastroliths. Without gastroliths, the longest dive recorded from all the alligators was 883 s (∼14.7 min), compared with 2122 s (∼35.4 min) with gastroliths. Without gastroliths, the shortest maximum dive recorded was 491 s (∼8.2 min) compared with 887 s (∼14.8 min) with gastroliths. Six of the seven alligators had a minimum duration of their dive that was longer with gastroliths than without gastroliths. The specific gravity of one juvenile that weighed 1 kg on land was found to be 1.04.

## Discussion

Water is both a refuge and a foraging habitat for crocodylians, and diving plays a central role in their ecology and physiology (Cambpell et al 2010; Kirshner 1985; Seebacher et al. 2005; Grigg and Kirshner 2015; Rodgers et al. 2015; Rodgers and Franklin 2017). Given the importance of diving to their life history, it is expected that positive selection has shaped diving behaviors, and lithophagy may be one facet of these behaviors. On the other hand, lithophagy could serve no function, and simply be a consequence of accidental ingestion of stones, or confusion of stones with food. In our study, four of seven alligators voluntarily ingested granite gastroliths weighing 2.5% of their body mass. The remaining three animals voluntarily ate a portion of the stones. While it has long been known that gastroliths are commonly found in the stomach of crocodylians, few empirical studies illuminate whether these stones are deliberately ingested and if they have a function. Davenport et al. (1990) offered one hatchling crocodile (*C. porosus* 300 g) a dish containing 10 small stones. After 24 h the dish was emptied of eight of the stones and X-rays confirmed that they had been ingested, but the impact of such stones on function or behavior was not determined.

The function of gastroliths in crocodylians has long been an enigma. Field data indicate that gastroliths may be more important for large individuals than for juveniles. In a study of wild Nile crocodiles, Cott (1961) did not find gastroliths in the smallest animals. However, Davenport et al. (1990) found gastroliths in all 12 hatchling *C. porosus* that had been shipped from a crocodile farm in Australia. An increase in specific gravity produced by gastroliths may be most important for large crocodylians that subdue their prey by dragging them underwater, in contrast to juvenile crocodylians, which tend to eat small prey, such as invertebrates. However, predation rates are very high in juvenile crocodylians and their predators include birds and other animals that seek them at the surface or on land (Woodward et al. 1987). Therefore, a behavior that enhances the ability of juvenile crocodylians to remain submerged may have high selective value. Furthermore, juveniles have cartilaginous tissues that have lower specific gravity than the bone that will replace these tissues in later life, and the rate of depletion of oxygen stores during diving is relatively higher in juveniles than in larger animals (Seebacher et al. 2005), making the increase in diving lung volume enabled by lithophagy potentially more important in juvenile animals than in adults. Wright and Kirshner (1987) showed that lung volume during dives in *C. porosus* scales logarithmically with body mass, with an exponent of 0.906, indicating mass-specific lung volumes decrease with body mass. Our study shows a significant effect on the duration of dives with the ingestion of gastroliths on juvenile alligators, but future studies of the relationship between lithophagy and body size are warranted.

Our experimental results indicate lithophagy prolongs the mean length of dives from about 6 to 11 min. The median of the mean dives with gastroliths, 10.4 min, (Fig. 1) is remarkably similar to the median duration of resting dives (∼11.5 min) measured in the wild, from somewhat larger (5–8 kg), freshwater crocodiles (*Crocodylus johnstoni*) at a similar temperature (Campbell et al, 2010). Our results are also consistent with Henderson's models of the effects of gastroliths on buoyancy (Henderson 2003). This model indicates that, if animals without gastroliths dive with the lungs half full of gas, gastroliths weighing 1–2% of the alligators body weight could increase the amount of air an animal could hold in its lungs by 20–40%, and still allow the animals to have a specific gravity greater than 1, and thus be able to sink passively. Experimental data indicate that lung volumes are generally less than 50% during voluntary dives in crocodylians (Wright and Kirshner 1987), and in this case gastroliths of 1–2% body mass could have a greater impact on lung oxygen stores.

Finally, consideration of specific gravity provides further perspective on the impact of lithophagy on diving in crocodylians. In our study, specific gravity of a 1 kg animal without air in the lung was estimated to be 1.04. This is similar to the value of 1.07 measured in adult human males (Behnke et al. 1942). In *C. porosus*, the specific gravity during diving is 1.02 (Wright 1985). If a 1 kg alligator dives with approximately the same lung volume as a juvenile *C. porosus*, the lungs would contain about 40 mL of gas (Wright and Kirshner 1987). Addition of 2.5% of body mass in granite gastroliths would require the animal to increase lung volume by 14.6 mL to maintain the same buoyancy, which is an increase in lung volume of about 36%. This increase in lung volume could lead to larger oxygen stores and thereby increase the average duration of dives.

## Funding

Funding was provided by Trinity College Dublin.

## Ethics statement

All experiments conformed to required regulatory standards.

## Competing interests

No competing interests declared.

## Supplementary Material

Supplementary Data
